# Effect of degalactosylated bovine glycoprotein formulations MAF and M сapsules on lymphopenia and clinical outcomes in hospitalized COVID-19 patients: a randomized clinical trial

**DOI:** 10.1186/s12879-024-09286-0

**Published:** 2024-05-23

**Authors:** Toshio Inui, Oksana Kruglova, Olga Martynenko, Kostiantyn Martynenko, Vadym Tieroshyn, Anatoliy Gavrylov, Kentaro Kubo, Hajime Yamakage, Borys Kutsyn, Alla Kubashko, Zoryana Veklych, Yurika Terashima, Martin Mette, Galyna Kutsyna

**Affiliations:** 1 Saisei Mirai Cell Processing Center, Osaka, Japan; 2 Saisei Mirai Keihan Clinic, Osaka, Japan; 3 Saisei Mirai Kobe Clinic, Kobe, Japan; 4 Saisei Pharma, Osaka, Japan; 5 Luhansk State Medical University, Rubizhne, Ukraine; 6 Central Hospital of Rubizhne, Luhansk Oblast, Ukraine; 7 Kharkiv National Medical University, Kharkiv, Ukraine; 8 Kyoto Medical Center, National Hospital Organization, Clinical Research Institute, Kyoto, Japan; 9 Armed Forces of Ukraine, Kyiv, Ukraine; 10 Shalimov’s National Institute of Surgery and Transplantation National Academy of Medical Science of Ukraine, Kyiv, Ukraine; 11 Kyiv National University named after Taras Shevchenko, Kyiv, Ukraine; 12 Graduate School of Medicine, Osaka Metropolitan University, Osaka, Japan; 13 Medicom clinic, Kyiv, Ukraine

**Keywords:** COVID-19 treatment, Saisei MAF capsules, Saisei M capsules, Lymphopenia, Mortality, Mucosal immunity

## Abstract

**Background:**

Targeting mucosal immunity of the gut, which is known to provide antigen processing, while avoiding excessive or unnecessary inflammation, was tested as a way to modulate COVID-19 severity.

**Methods:**

Randomized open-label trial in 204 adults hospitalized with non-critical COVID-19 who received for 14 days in addition to standard of care (SOC) degalactosylated bovine glycoproteins formulations of either MAF capsules (MAF group) or M capsules (M group) or SOC only (control group).

**Results:**

Median recovery time when patients did not require supplemental oxygen was 6 days in both study groups compared to 9 days in the control (MAF vs. control; *P* = 0.020 and M vs. control; *P* = 0.004). A greater reduction in mortality was seen in the MAF group compared to the control by day 14 (8.3% vs. 1.6%; *P* = 0.121) and by day 29 (15.3% vs. 3.2%; *P* = 0.020), and similarly in the M group by day 14 (8.3% vs. 2.9%; *P* = 0.276) and by day 29 (15.3% vs. 2.9%; *P* = 0.017). The proportion of those who had baseline absolute lymphocyte count (ALC) lower than 0.8 × 10^9^/L was 13/63 (20.6%), 17/69 (24.6%), and 18/72 (25.0%) of patients in MAF, M, and control group respectively. Day 29 mortality among these lymphopenic patients was three times higher than for the intent-to-treat population (21% vs. 7%) and consisted in above subgroups: 2/13 (15%), 2/17 (12%), and 6/18 (33%) of patients. The decreased mortality in both study subgroups correlated with greater ALC restoration above 0.8 × 10^9^/L level seen on day 14 in 91% (11/12) and 87.5% (14/16) of survivors in MAF and M subgroups respectively compared to 53.3% (8/15) of survivors in control subgroup. Incidences of any ALC decrease below the baseline level on day 14 occurred in 25.4% of patients in the MAF group and 29.0% of patients in the M group compared to 45.8% in control and ALC depletion by ≥ 50% from the baseline level consisted of 7.9%, 5.8%, and 15.3% of cases in these groups respectively.

**Conclusion:**

This study showed that both study agents prevented ALC depletion and accelerated its restoration, which is believed to be one of the mechanisms of improved crucial clinical outcomes in hospitalized COVID-19 patients.

**Trial registration:**

The trial was registered after the trial start in ClinicalTrials.gov NCT04762628, registered 21/02/2021, https://www.clinicaltrials.gov/ct2/show/NCT04762628.

**Supplementary Information:**

The online version contains supplementary material available at 10.1186/s12879-024-09286-0.

## Introduction

MAF capsules and M capsules are dietary supplements produced by Saisei Pharma, Japan. They are designated to modulate the mucosal immunity of the intestine. The main active ingredients of both products are vitamin D binding protein (VDBP) and other glycoproteins which undergo degalactosylation during the process of β-Galactosidase treatment applied to the whole heat-inactivated bovine colostrum in the case of MAF Capsules and to bovine whey in the case of M Capsules. This treatment converts VitD ∼ VDBP into VitD-degalactosylated VDBP. The functional activity of degalactosylated VDBP is similar to that seen in the group-specific component macrophage activating factor (GcMAF). GcMAF is a protein that results from the sequential deglycosylation of its precursor - VDBP. The group-specific component (Gc) protein - VDBP is produced in the liver and present in the majority of biological fluids. It has multifunctional properties as a transporter of serum vitamin D3 and its metabolites, functions as an actin scavenger during cellular injury, acts as a chemotaxin for phagocytic cells, and also plays a role in macrophage activation as a precursor for GcMAF. Gc protein has a triple-domain modular structure, where Domain III (C-terminal end) harbors a single glycosylation site [1]. The terminal N-acetylgalactosamine (GalNAc) moiety in domain III is the region involved in the GcMAF-mediated macrophage activation cascade. During inflammation, lysophosphatidylcholine is released from tissue which induces the expression of beta-galactosidase in B cells and sialidase in T cells. These enzymes hydrolyze Gc protein’s terminal galactose and sialic acid saccharides to convert it into GcMAF with an N-acetylgalactosamine moiety [1,2]. This process can be simulated by exposing Gc protein-containing biological fluids such as bovine colostrum and whey, and human serum to beta-galactosidase and sialidase treatment [3]. However, in vitro studies showed that bovine colostrum can acquire similar macrophage activation potency after treatment with β-Galactosidase alone. The studies showed that an increase in the phagocytic activity of mouse peritoneal macrophages induced by degalactosylated bovine colostrum was only slightly less than that seen with degalactosylated/desialylated bovine colostrum [4]. Bovine colostrum and bovine whey glycoproteins, including Gc protein, which lack galactose NAc, can undergo further cleavage of terminal sialic acids by resident sialidases in the small intestine which converts degalactosylated Gc protein into GcMAF. It has also been shown that the Gc1f1f protein lacking galactose (preGc1f1fMAF), can be converted to GcMAF in vivo by resident sialidase of mouse peritoneal fluid (http://ar.iiarjournals.org/content/32/6/2359.long). The other degalactosylated Galactose (Gal) and N-acetylgalactosamine (GalNAc) glycans contained in bovine colostrum and bovine whey glycoproteins are also expected to increase their immunomodulatory activity and contribute to the functional activity of both products. Both study products use acid-resistant capsules which are designed to release their contents of galactose NAc-containing glycoproteins, including Gc protein, in the target gut’s mucosal immunity site. This is where they have to reveal their highest macrophage activation potency after cleavage of terminal sialic acids by resident sialidases resulting in degalactosylated Gc protein converted into GcMAF. One of the targeted cells there are resident intestinal macrophages with low expression of innate response receptors, which recognize and process antigens in a tolerizing manner and exhibit great phagocytic and bactericidal activity without initiating an inflammatory responses. These constitute the largest pool of macrophages in the body. They serve the function of protecting against unwanted immune responses and can down-regulate an excessive systemic inflammatory response, contributing to the resolution of inflammation and inducing tolerance to foreign antigens, as well as autoantigens 10.1172/JCI19229.

M capsules and MAF capsules are potential immunomodulators that can increase antigen processing and the capacity of macrophages to resolve inflammation and modulate the mucosal immune response in the small intestine in conditions of non-critical COVID-19.

This trial was initiated in Ukraine in October 2020 and terminated in June 2021. The study interim analysis included 204 patients who were enrolled before the enrolment was interrupted in June 2021 due to dramatically declining hospitalized cases. Based on the interim analysis result the study has been terminated earlier as effective. Here, we describe the crucial endpoints and the analysis of the data until study day 29 obtained on 204 enrolled subjects, in which we evaluate the treatment with MAF and M capsules compared to the control.

## Methods

### Design

This is an adaptive, open-label, multicenter, proof-of-concept randomized clinical trial. Enrolment in this trial began in October 2020 and ended in June 2021. There were 2 trial sites in Ukraine. Eligible patients were randomly assigned in a 1:1:1 ratio to receive either SOC only (control group), or MAF capsules (MAF group), or M capsules (M group) in addition to SOC.

Randomization was stratified by age, a known as one of the key factors in COVID-19 disease severity. A sequential block-permuted randomization design was employed to assign participant to one of the study groups. Randomization lists were prepared separately for each clinical site. Eligible study participants were first stratified by age: less than 60 years old and 60 years or older. Further, participants were randomly allocated to study groups within each stratum in a 1:1:1 ratio.

MAF capsules and M capsules were administered orally as a 148 mg dose three times daily for 14 days. Study products intake interruption was prespecified in cases of applied mechanical ventilation or swallowing impairment for any reason. If such an event continued for ≤ 5 days, the rest of the treatment course would be taken starting from the day when the ability for oral capsule intake was restored. In case mechanical ventilation or swallowing impairment continued for more than 5 days, the study product treatment was not resumed, and the subject was to be followed up till death or the end of the study. The SOC group was used as a control in this open-label trial. All patients received SOC according to the actual Ukrainian recommendations/guidelines regarding the treatment of COVID-19. The trial protocol was approved by the ethics committee at each site. Written informed consent was personally obtained from each patient.

The two study clinical sites were implemented within a network of hospitals that collect data using the ISARIC-World Health Organization Clinical Characterization Protocol and data tools for COVID-19 patients. Clinical sites adhered to the standardized in ISARIC COVID-19 study laboratory units, clinical and laboratory data collection algorithms, gathering data through an international electronic data capture system, contributing to the formation of global databases with prospectively collected clinical data on individuals hospitalized with COVID-19 (https://isaric.org/). This electronic database facilitated remote, real-time monitoring of the captured study data. The electronic Case Report Form (eCRF) for study participants includes various modules: the enrolment module, day 1, 7, 14, and 29 study treatment visits modules, intensive care treatment, and discharge. The reported during acute COVID-19 treatments data encompass a wide range of information, including signs and symptoms, pre-existing comorbidities, anthropometric data, vital signs, chronic and acute treatments, study treatments, complications, laboratory data, dates of hospitalization and discharge, mortality, and vaccination status. In addition to the eCRF modules, a paper version CRF was utilized. It included all the modules listed above, along with the daily monitoring of vital signs (blood pressure, respiratory rate, body temperature, oxygen saturation in capillary blood (SpO2%)), and daily changes in the COVID-19 WHO 8 score ordinary scale, treatment tolerability, and adverse reactions.

In addition to the information presented here, further details about the study’s methods are available in the Supplemental Materials file titled “Randomization, Data Collection, and Statistical Analyses”.

### Procedures

Study subjects were assessed daily while hospitalized, from day 1 through day 29. During hospitalization, patients’ clinical status was assessed using the WHO 9-point Ordinal Scale for Clinical Improvement. The study has treatment visits on days 1, 7, and 14, and posttreatment follow-up on days 29 and 60. Those subjects who were discharged from the hospital before day 14, had this visit as outpatients. Safety laboratory tests were obtained on days 1 (prior to study treatment), days 7, and 14. All serious adverse events and grade 3 or 4 adverse events that showed an increase in severity from baseline and grade 2 or higher suspected study products related hypersensitivity reactions were recorded.

### Patients

Hospitalised patients were at least 18 years of age with SARS-CoV-2 infection confirmed by polymerase chain reaction (PCR). Patients had a respiration rate of ≤ 29 per minute and oxygen saturation (SpO2) of ≤ 95% on room air, with respiratory symptoms appearing not more than 7 days before enrolment. Patients were excluded if they were receiving immunosuppressive or other immune-based therapy such as COVID-19 convalescent plasma, immunoglobulin products, or interferons at entry. Patients requiring mechanical ventilation and ICU admission at screening were excluded.

### Main outcomes

The first primary outcome was the time to basic clinical improvement and to recovery, defined as the first day, during the 29 days after enrolment, on which a patient did not require any oxygen therapy or hospitalization, and the proportion of patients limited in activity after recovery. The second primary outcome was mortality for any reason on days 14 and 29 since the study treatments started.

The secondary outcomes were the incidence and duration of new noninvasive ventilation or high-flow oxygen and invasive ventilation up to day 29. Another secondary outcome was the time to the improvement of one category and of two categories from the baseline ordinal score; clinical status on the ordinal scale on day 14. The categories are as follows: 8. Death; 7. Hospitalized, on invasive mechanical ventilation with vasopressor or Extracorporeal Membrane Oxygenation; 6. Hospitalized, on invasive mechanical ventilation; 5. Hospitalized, on non-invasive ventilation or high-flow oxygen devices; 4. Hospitalized, requiring low-flow supplemental oxygen; 3. Hospitalized, not requiring supplemental oxygen - requiring ongoing medical care (coronavirus (COVID-19) related or otherwise; 2. Not hospitalized, limitations on activities and/or requiring home oxygen; 1. Not hospitalized, no limitations on activities; 0. No clinical or virological evidence of infection. Secondary safety outcome measures included grade 3 and 4 adverse events and serious adverse events that occurred during the trial, discontinuation or temporary suspension of study product intake and changes in assessed laboratory values over time.

## Results

### Patients

Of the 235 patients who were assessed for eligibility, a total of 204 patients underwent randomization with 63 assigned to MAF Capsules, 69 to M Capsules, and 72 to control (intention-to-treat population). The study inclusion criteria allowed patients with respiration rates ≤ 29 per minute and SpO2 ≤ 95% on room air to be included. The mean time between symptom onset and randomization was 5 days. All enrolled patients on baseline had clinical signs of low respiratory tract involvement and pneumonia was confirmed in all of them by chest radiography or computed tomography during the next one-three days of hospitalization. Based on the last WHO classification a total of 183 (89.7%) were categorized as having moderate disease with SpO2 ≥ 90% on room air and 21 (10.3%) as having severe disease. A total of 19 patients (9.3%) met category 5 criteria on the ordinal scale, 183 (89.7%) category 4, and 2 (1%) category 3 at enrolment (Table 1). 35.8% of the patients were male. The patients were in the 38–90 years age range. The mean age of patients was 63.5, 63.6, and 63.6 years in the MAF group, M group, and control group respectively (Table 1). Most patients had either one or two or more of the coexisting comorbidities at enrolment, and most commonly this was hypertension and chronic heart disease, chronic neurological disorders, and type 2 diabetes mellitus. While stratifying randomization by age, a known cumulative prognostic factor for acute COVID-19 mortality, effectively balances groups for this covariate, it may inherently allow other baseline characteristics to remain imbalanced. We used absolute standardized differences (ASD) to assess their balance between groups. The baseline characteristics, including sex, age, comorbidities, baseline ordinary score (3, 4, or 5), SpO2 level (≥ 90% vs. <90%), and lymphopenia status, were considered to be in balance between study groups with ASD values less than 0.25. This suggests that three study groups were well-matched with respect to these factors, reducing the potential for confounding variables to influence the study results (Table 1).

Of the patients assigned to receive MAF capsules, 61 patients (96.8%) received them as assigned, and of those assigned to receive M capsules, 67 patients (97.1%) received them as assigned. No patients had both study agents intake discontinued before day 14 because of an adverse event or had a serious adverse event other than death, nor did any patients in the study groups withdraw their consent.

A total of 62 patients in the MAF group, 69 patients in the M group, and 70 patients in the control group completed the trial through to the day 29, recovered, or died; one patient in the MAF group and two patients in the control group passed the visit on day 14 but did not come on the scheduled day 29 visit after discharge from the hospital. Their surveillance status was confirmed by phone call. The as-treated population included 204 patients who received the assigned treatment (63 assigned to the MAF group, 69 to the M group, and 72 to the control group).

**Table 1:** Clinical Characteristics of the Patients at Baseline

	Control *N* = 72		MAF *N* = 63		M *N* = 69		ASD*
**Characteristic**							
Male sex — no. %	26	36.1%	25	39.4%	22	31.9%	0.157
Age– years							0.007
Mean ± SD	63.6 ± 10.7		63.5 ± 10.5		63.6 ± 10.7		
Median (IQR)	65.0 (56.0, 72.0)		65.0 (56.0, 71.0)		64.0 (57.5, 70.5)		
Range (min-max)	38.0–87.0		34.0–83.0		38.0–90.0		
**Chronic Comorbidities — no. %**							
Heart diseases	55	76.4%	49	77.8%	51	73.9%	0.091
Hypertension	53	73.6%	47	74.6%	48	69.6%	0.112
Neurological disorders	25	34.7%	21	33.3%	16	23.2%	0.246
Type 2 diabetes	19	26.4%	13	20.6%	16	23.2%	0.136
Smoking-induced COPD	7	9.7%	11	17.5%	13	18.8%	0.227
**Baseline ordinary score — no. %**							0.144
3. Not requiring supplemental oxygen	0	0%	2	3.2%	0	0%	
4. Requiring low flow oxygen	66	91.7%	55	87.3%	62	89.8%	
5. Requiring non-invasive ventilation or high flow oxygen	6	8.3%	6	9.5%	7	10.1%	
**SpO2 level — no. %**							0.053
SpO2 ≥ 90%	64	88.9%	57	90.5%	62	89.8%	
SpO2 less than 90%	8	11.1%	6	9.5%	7	10.1%	
**Baseline lymphopenia— no. %**							0.055
ALC less than 1.0 × 10^9^/L	30	41.7	28	44.4	29	42.0	
ALC less than 0.8 × 10^9^/L	18	25.0	13	20.6	17	24.6	

*Abbreviations* SD - Standard deviation; IQR - Interquartile Range, ALC - Absolute Lymphocyte Count, COPD - Chronic Obstructive Pulmonary Disease, ASD: absolute standardized difference

*An ASD values less than 0.25 used to indicate good balance between the study groups

At both study clinical sites SOC was adhered to national guideline for hospitalized COVID-19 patients in all treatment groups. The national guidelines for the COVID-19 SOC largely aligned with the WHO recommendations at the time of the study. The concurrent use of any other experimental treatments, off-label drugs, or interventions intended for specific treatment of COVID-19 or SARS-CoV-2 infection was prohibited. Remdesivir was a component of SOC, while other antiviral drugs were not included. Hydroxychloroquine was part of SOC for patients with severe and critical disease to manage cytokine storm syndrome. However, remdesivir and hydroxychloroquine were limited and just a few patients received these drugs. Systemic corticosteroid therapy was administered to all patients with severe and critical COVID-19 and conditionally to patients with non-severe COVID-19, among them, those with high levels of inflammatory markers and an increased need for supplemental oxygen, and other signs of respiratory deteriorations. Thromboprophylaxis during the hospital stay mainly included prophylactic-dose of Low-molecular-weight heparin.

During the study, 70.8% of patients in the control group, 60.3% in the MAF group, and 66.7% in the M group received antibiotics due to secondary bacterial co-infections such as bacterial pneumonia. Antifungal therapy was administered in 37.5%, 34.9%, and 39.1% of patients in the control, MAF, and M group respectively. Remdesivir was administered in 4.2%, 6.4%, and 2.9% of patients in control, MAF, and M group respectively. Glucocorticoids were administered on day 1 in 27.8%, 30% and 26.1%, and later during the study in 40.3%, 22.4%, and 30.4% of patients in control, MAF and M group respectively. The mean duration of glucocorticoid administration was 10.6, 9.7, and 9.4 days in the control, MAF, and M group respectively (Table 2).

**Table 2:** Standard of Care of COVID-19 applied in three study groups

	Control *N* = 72		MAF *N* = 63		M *N* = 69	
No. of events/% from total patients no.	N	%	N	%	N	%
Heparin Low-molecular-weight	71	98.6	60	95.2	67	97.1
Remdesivir	3	4.2	4	6.4	2	2.9
Hydroxychloroquine					3	4.3
Antibiotics	51	70.8	38	60.3	46	66.7
Antifungals	27	37.5	22	34.9	27	39.1
Dexamethasone	49	68.1	33	52.4	39	56.5
Mean duration of the course in days	10.6		9.7		9.4	

## Primary outcomes

### Clinical improvement and recovery

Among the 202 patients receiving oxygen at enrolment, those alive on day 29 in the MAF and M groups had a shorter time to basic improvement when they did not require any more supplemental oxygen than patients in the control group (median, 6 days in the MAF group compared to 8 days in the control group; *P* = 0.030, median, 6 days in M group compared to 8 days with the control group; *P* = 0.006) (Table 3).

Patients in the MAF group had a shorter time to discharge than those in the control group (median, 13 days vs. 14 days; *P* = 0.064). Patients in the M group had a significantly shorter time to discharge than those in the control group (median, 13 days vs. 14 days; *P* = 0.017) (Table 3).

The proportion of those discharged without limitations on their activities was greater in the MAF group 55.5% and in the M group 50.7%, compared to 29.2% in the control group (Table 4). After discharge, no one patient received supplemental oxygen.

### Mortality

In the intent-to-treat population the hospital mortality was 4.4% by day 14, 7.4% by day 29, and total hospital mortality through day 34 was 7.8%. Mortality by day 14 was 1.6% in the MAF group, 2.9% in the M group, and 8.3% in the control group, and mortality by day 29 was 3.2%, 2.9%, and 15.3% in these groups respectively. Fisher’s exact estimates of the reduction in mortality in the MAF group vs. control group by day 14 (*P* = 0.121) and significant reduction by day 29 (*P* = 0.020) and in the M group vs. control group by day 14 (*P* = 0.276) and by day 29 (*P* = 0.017).

**Fig. 1:**
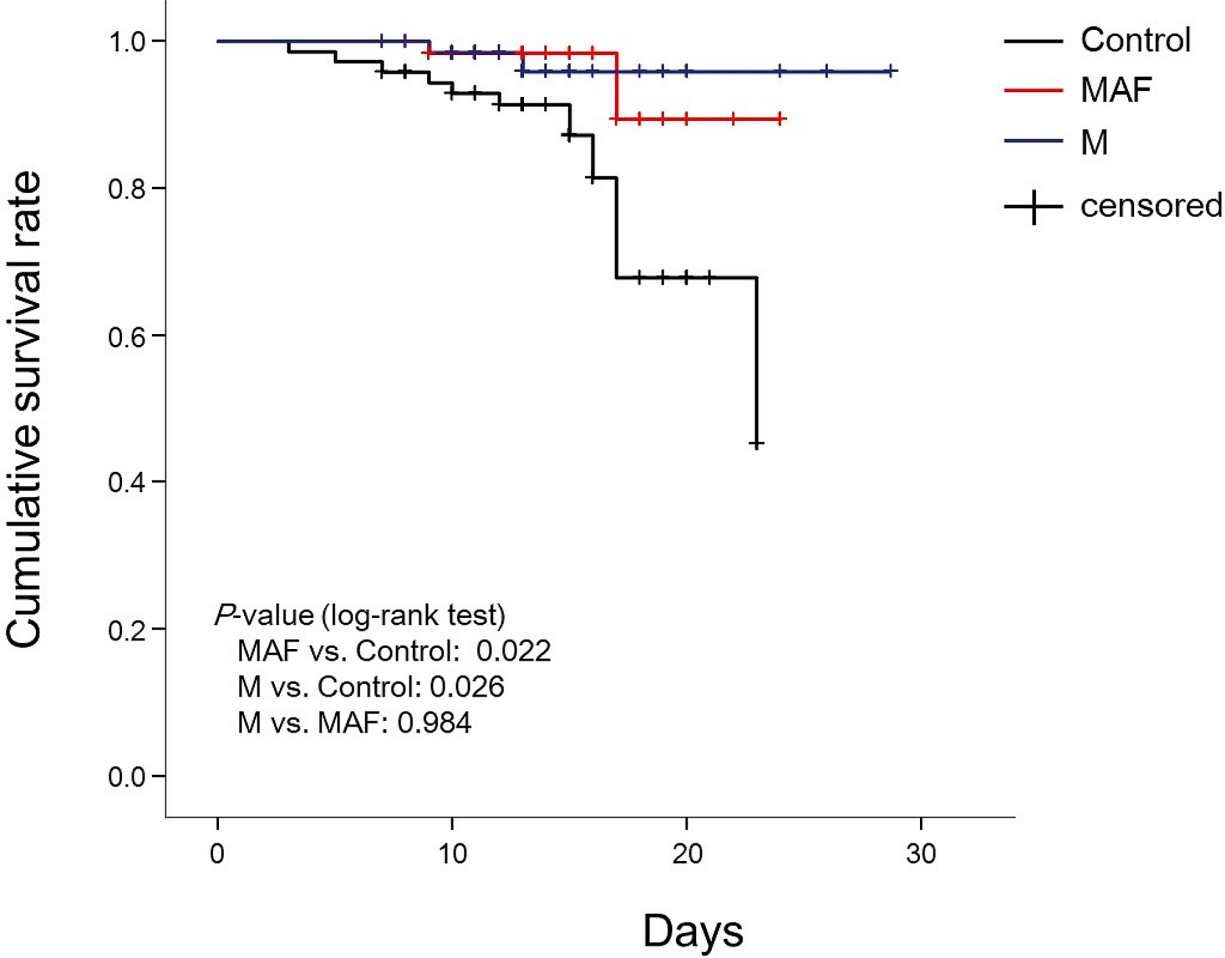
Kaplan-Meier curve comparing survival of groups MAF, M, and control Focusing on 29-day mortality as one of the primary outcomes, each line on the Kaplan-Meier curve depicts the estimated probability of surviving over 29 days from study treatment started for each group. The log rank test used to compare the survival times between three study groups

29-day survival analysis was performed using the Kaplan-Meier method with death from any cause as the outcome and the number of days from the start of study treatment to the occurrence of death as the survival time (Fig. 1). The log-rank test used for cumulative survival analysis indicates a statistically significant difference in survival time between the MAF and M groups compared to the control group (*p* = 0.022, for the first and *p* = 0.026 for the second comparison). This means that the survival times are significantly longer in both study groups compared to the control group. There is no significant difference in survival time between the MAF and M groups.

There was no correlation between mortality and co-existing pathology due to the relatively small study cohort and patients’ uniformity, as the mean age of patients was 63.5, 63.6, and 63.6 in the three studied groups and it was linked with the common Ukrainian population comorbidities in this age category (Table 1). There also was no correlation of mortality with COVID-19 severity status at enrolment, last was mainly defined by baseline SpO2 (Tables 1 and 5). However, the positive correlation of ALC low on baseline or declined later with respect to mortality was seen (Table 5), which is described in the Absolute lymophocyte count section.

### Secondary outcomes

202 out of 204 enrolled patients received either low-flow or high-flow oxygen or non-invasive ventilation oxygen at enrolment (Table 1), and for the remaining two patients in the MAF group the low-flow oxygen was administered in the first two days after enrolment. Alive on day 29 patients in the MAF group and the M group continued to receive oxygen for fewer days than patients in the control group (median, 6 days for the MAF group vs. 8 days for the control group; *P* = 0.030 and median 6 days for the M group vs. 8 days for the control group; *P* = 0.006) (Table 3).

Among 185 patients who were not receiving noninvasive ventilation, high-flow oxygen, invasive ventilation, or ECMO at baseline, the incidence of new noninvasive ventilation or high-flow oxygen use was lower in the MAF group than in the control group (10.5% vs. 16.7%) and it was lower in the M group than in the control group (6.5% vs. 16.7%) (Table 3). Duration of noninvasive ventilation or high-flow oxygen among patients who were receiving these interventions at enrolment and during the study was similar in the MAF group and the control group and was fewer in one subsequent day in the M group than those in the control group (median, 4 days vs. 5 days; *P* = 0.444) (Table 3).

No patients from the intent-to-treat population received mechanical ventilation at enrolment, and the incidence of this intervention use during the study was lower in the MAF group than in the control group (3.2% vs. 12.5%; *P* = 0.061) and was significantly lower in the M group than in the control group (1.4% vs. 12.5%; *P* = 0.018).

Among the 204 enrolled patients, none were admitted to the Intensive Care Unit (ICU) on day 1, and the respiratory deteriorations and other life-threatening conditions ratio that required admission to ICU were lower during the study in the MAF group than in the control group (9.5% vs. 16.7%; *P* = 0.311) and significantly lower in the M group than in the control group (4.3% vs. 16.7%; *P* = 0.027) (Table 3).

**Table 3:** Overall Outcomes in the Intention-to-Treat Population

	Control *N* = 72	MAF *N* = 63	M *N* = 69	P-value (vs. control)	
				MAF	M
**Duration hospitalisation, days**					
Mean ± SD	13.9 ± 3.8	13.7 ± 3.4	13.7 ± 4.1		
Median [IQR]	14.0 [13.0, 15.0]	13.0 [12.0, 15.0]	13.0 [12.0, 14.0]	0.166	0.056
Range (min - max)	2.0–23.0	8.0–24.0	7.0–34.0		
**Among those who were alive on day 29**					
Mean ± SD	14.2 ± 3.1	13.8 ± 3.4	13.4 ± 3.3		
Median [IQR]	14.0 [13.0, 15.0]	13.0 [12.0, 15.0]	13.0 [12.0, 14.0]	0.064	**0.017**
Range (min - max)	7.0–23.0	8.0–24.0	7.0–26.0		
**Oxygen**					
**Total oxygen therapy days in intent-to-treat population**					
Mean ± SD	9.9 ± 5.1	7.9 ± 5.2	7.8 ± 5.8		
Median [IQR]	9.0 [5.3, 13.0]	6.0 [4.0, 11.0]	6.0 [4.0, 10.5]	**0.020**	**0.004**
Range (min - max)	2.0–23.0	0.0–24.0	1.0–33.0		
**Total oxygen therapy days among those who were alive on day 29**					
Mean ± SD	9.5 ± 4.9	7.8 ± 5.1	7.4 ± 4.9		
Median [IQR]	8.0 [5.0, 12.0]	6.0 [4.0, 10.5]	6.0 [3.8, 9.3]	**0.030**	**0.006**
Range (min - max)	2.0–21.0	0.0–24.0	1.0–22.0		
**Noninvasive ventilation or high-flow oxygen**					
Applied at baseline (No. of events/total patients no. %)	6/72 8.3%	6/63 9.5%	7/69 10.1%		
New use (No. of events/total patients no. %)	11/66 16.7%	6/57 10.5%	4/62 6.5%	0.434	0.099
Duration days, median [IQR]	5.0 [4.0, 10.0]	5.0 [5.0, 6.0]	4.0 [3.0, 7.0]	0.733	0.444
**New use of invasive ventilation (No. of events/total patients no. %)**	9/72 12.5%	2/63 3.2%	1/69 1.4%	0.061	**0.018**
Duration days, median [IQR]	2 [1.0, 2.0]	3 [2.0. 4.0]	6 days/ 1 event	0.436	n/d§
**ICU admission (No. of events/ total patients no. %)**	12/72 16.7%	6/63 9.5%	3/69 4.3%	0.311	**0.027**
**Mortality**					
Through day 14‡ (No. of events/total patients no. %)	6/72 8.3%	1/63 1.6%	2/69 2.9%	0.121	0.276
Through day 29‡ (No. of events/total patients no. %)	11/72 15.3%	2/63 3.2%	2/69 2.9%	**0.020**	**0.017**
Through day 29 in subgroups BL ALC lower 0.8 × 10^9^/L (No. of events/total patients no. %)	6/18 33%	2/13 15.4%	2/17 11.8%	0.412	0.228
Total hospital mortality through day 34 (No. of events/total patients no. %)	11/72 15.3%	2/63 3.2%	3/69 4.3%	**0.020**	**0.046**

*Abbreviations* BL ALC, Baseline Absolute Lymphocyte Count

‡ Mortality over the first 14 days includes data from all patients who were still alive through 14 days post-enrolment, with data censored on day 15. Mortality over the 29 days uses the totality of the study data and censors’ data from patients who completed follow-up alive at 29 days post-enrolment

P-value: Fisher’s exact test [No correction for multiplicity], n/d - not detected meaning of p - confidence factor

### Clinical status on ordinal score at day 14

At day 14 after enrolment, 87.3% in the MAF group, 86.9% in the M group versus 73.6% of patients in the control group reached one of the primary recovery endpoints: when they did not require supplemental oxygen till being hospitalized or discharged from the hospital. Among day 14 recovery cohorts, the proportion of total discharged from the hospital was 63.4%, 66.6%, and 59.7% in the MAF group, M group, and control group respectively; as compared to the control group, the proportion of those discharged without limitations on their activities was greater in the MAF group (55.4% vs. 29.2%; *P* = 0.03) and also greater in the M group (50.7% compared to 29.2%; *P* = 0.01) (Table 4). After discharge, no patients received supplemental oxygen and the limitations on their activities were mainly associated with post-COVID-19-related fatigue and mild to moderate signs of neurological disorders.

The day 14 mortality ratio was 1.6%, 2.9%, and 8.3% in the MAF group, M group, and control group respectively. On day 14, no patients required mechanical ventilation in the M group and it was applied for 1.6% of patients in the MAF group, compared to 7% of patients in the control group (Table 4).

**Table 4:** Outcomes According to Score on the Ordinal Scale in the Intention-to-Treat Population at day 14^ß^

	Control *N* = 72	MAF *N* = 63	M *N* = 69	P-value MAF vs. control	P-value M vs. control
**No. of events/% of total**					
0	2/2.8%	14/22.2%	10/14.5%		
1	19/26.4%	21/33.3%	25/36.2%		
2	22/30.6%	5/7.9%	11/15.9%		
3	10/13.9%	15/23.8%	14/20.3%		
4	6/8.3%	6/9.5%	6/8.7%		
5	2/2.8%		1/1.4%		
6	2/2.8%	1/1.6%			
7	3/4.2%				
8	6/8.3%	1/1.6%	2/2.9%		
**No. of events met primary criteria/% of total**					
Categories 0 + 1 + 2 + 3	53/73.6%	55/87.3%	60/86.9%	0.054	0.058
Categories 0 + 1 + 2	43/59.7%	40/63.4%	46/66.6%	0.724	0.485
Categories 0 + 1	21/29.2%	35/55.5%	35/50.7%	**0.003**	**0.010**

ß The ordinal score at day 14 is the patient’s worst score on the ordinal scale during the previous day. Scores on the ordinal scale are as follows: 8. Death; 7. Hospitalized, on invasive mechanical ventilation with vasopressor or Extracorporeal Membrane Oxygenation; 6. Hospitalized, on invasive mechanical ventilation; 5. Hospitalized, on non-invasive ventilation or high-flow oxygen devices; 4. Hospitalized, requiring low-flow supplemental oxygen; 3. Hospitalized, not requiring supplemental oxygen - requiring ongoing medical care (coronavirus/COVID-19 related or otherwise); 2. Not hospitalized, limitations on activities and/or requiring home oxygen; 1. Not hospitalized, no limitations on activities; 0. No clinical or virological evidence of infection

### Absolute lymphocyte count

The admission (day 1) median ALC value in the intent-to-treat population was balanced between groups and close to the lower limit of the normal range consisting of 1.12 [95% CI, 0.94 to 1.30], 1.24 [95% CI, 1.07 to 1.41], and 1.26 [95% CI, 1.09 to 1.42] in the MAF group, M group, and control group respectively. The normal range used for ALC was 1.10–4.00х10^9^/L. On day 7, median ALC increased significantly by 26% in the MAF group and by 16% in the M group, compared to the insignificant increase of 11% in the control group. By day 14, the increase was 51% in the MAF group, 44% in the M group, and 37% in the control (Table 6).

In the intent-to-treat population, 87 patients (42.6%) had baseline ALC levels lower than 1.0 × 10^9^/L, and 48 patients (23.5%) had levels lower than 0.8 × 10^9^/L. These patients were evenly distributed among the three study groups, forming related subgroups with initial lymphopenia (Tables 7 and 8). Hospital mortality has been linked to initial lymphopenia, as among a total of 16 mortality cases 13 (81.2%) had ALC levels lower than 1.0 × 10^9^/L at admission (Table 5). A high risk of COVID-19 deterioration was among subgroups of patients with profound lymphopenia on admission (ALC < 0.8 × 10^9^/L), leading to a day 29 mortality of 20.8%, nearly three times higher than the overall population (7.35%). However, mortality was lower in the MAF and M subgroups (15.4% (2/13) and 11.8% (2/17), respectively) compared to the control subgroup (33.3% (6/18)) (Table 3).

**Fig. 2:**
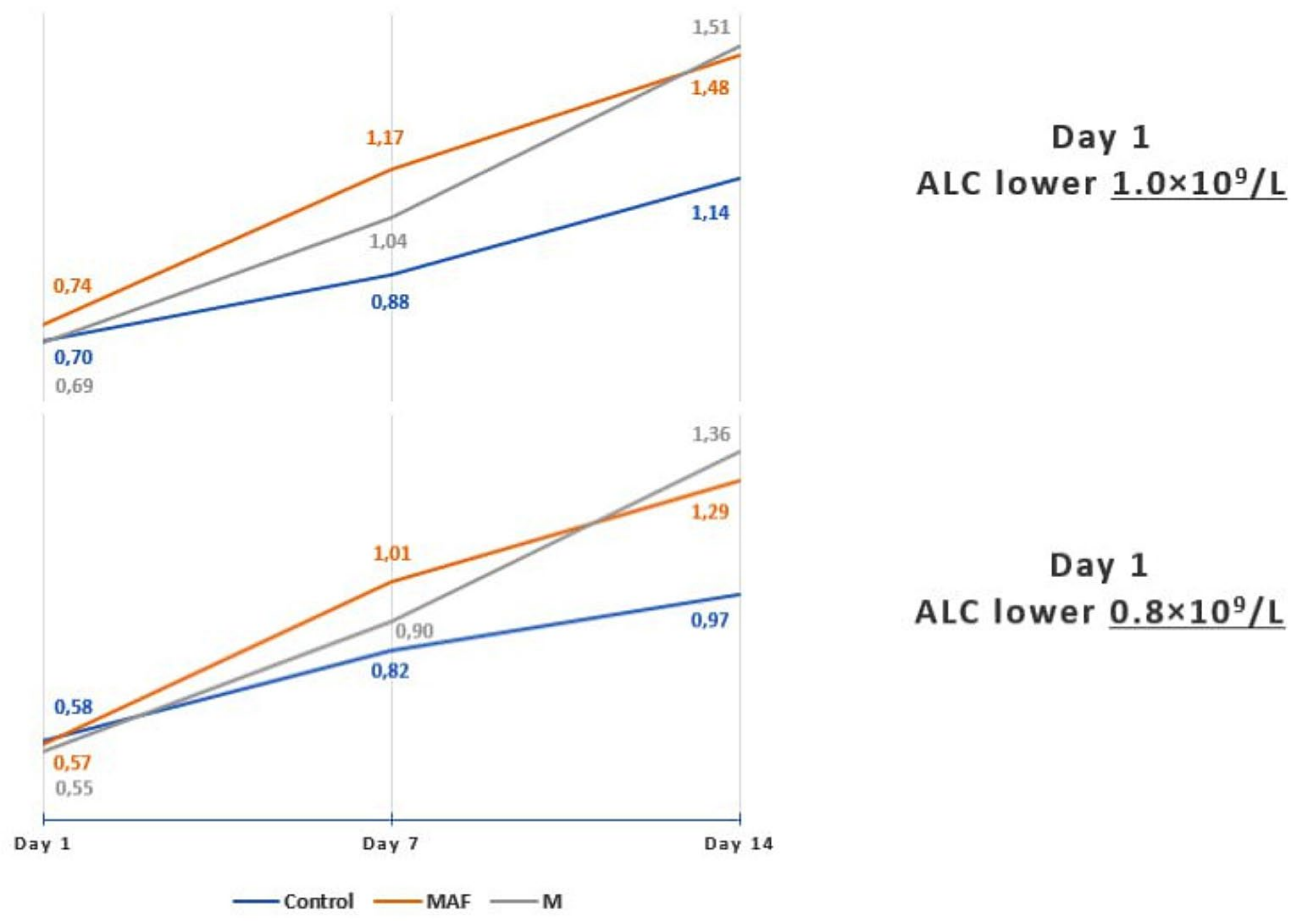
Changes in mean ALC values in 10^9^ cells/L over a two-week treatment period in subgroups of participants with lymphopenia at enrolment

Either the number of patients in the lymphopenic subgroups decreased during the study due to mortality, or there was a restoration in their ALC. After excluding mortality cases, we estimated the proportion of patients whose ALC remained below and above the indicated thresholds among survivors on day 7 and day 14 in subgroups with initial lymphopenia. This assessment aimed to evaluate the study treatments’ impact on ALC restoration across varying degrees of lymphopenia severity (Table 7; Fig. 2).

In subgroups of patients with baseline ALC lower than 1.0 × 10^9^ cells/L, ALC did not restore above 1.0 × 10^9^/L on day 7 and day 14, respectively, in 32.1% (9 out of 28) and 22.2% of patients (6 out of 27) in the MAF subgroup, and 55.2% (16 out of 29) and 28.6% of patients (8 out of 28) in the M subgroup, compared to 67.9% (19 out of 28) and 46.2% of patients (12 out of 26) in the control subgroup. This was where the most evident superiority of study treatments was observed, with twice as few patients failing to reach the targeted ALC recovery on day 7 in the MAF subgroup compared to the control (32.1% vs. 67.9%; *P* = 0.008). The proportion of patients whose ALC recovered above 1.0 × 10^9^/L level was higher in the MAF and M subgroups, comprising of 67.9% and 44.8% respectively, compared to 32.1% in the control subgroup on day 7, and 77.8% and 71.4% compared to 53.8% in these subgroups respectively on day 14 (Table 7; Fig. 2).

In subgroups of patients with profound lymphopenia (baseline ALC below 0.8 × 10^9^/L), the proportion of those whose ALC recovered above the 0.8 × 10^9^/L level was higher in the MAF and M subgroups (53.8% (7/13) and 64.7% (11/17), respectively) compared to the control subgroup (52.9% (9/17)) on day 7. On day 14, these proportions increased to 91.7%, 87.5%, and 53.3% in these subgroups, respectively. It resulted in a significantly lower proportion, compared to the control, in both the MAF subgroup (8.3% or 1/12 vs. 46.7% or 7/15; *P* = 0.025) and the M subgroup (12.5% or 2/16 vs. 46.7% or 7/15; *P* = 0.046) of patients whose ALC remained unrecovered above 0.8 × 10^9^/L (Table 7; Fig. 2). Consequently, by the end of the study treatments, on day 14, the proportion of vulnerable in terms of mortality patients with ALC below 0.8 × 10^9^/L was 5.6 times less in the MAF subgroup and 3.8 times less in the M subgroup compared to the relative control. The profound lymphopenia study cohort appeared to be the most responsive to both study treatments.

**Fig. 3:**
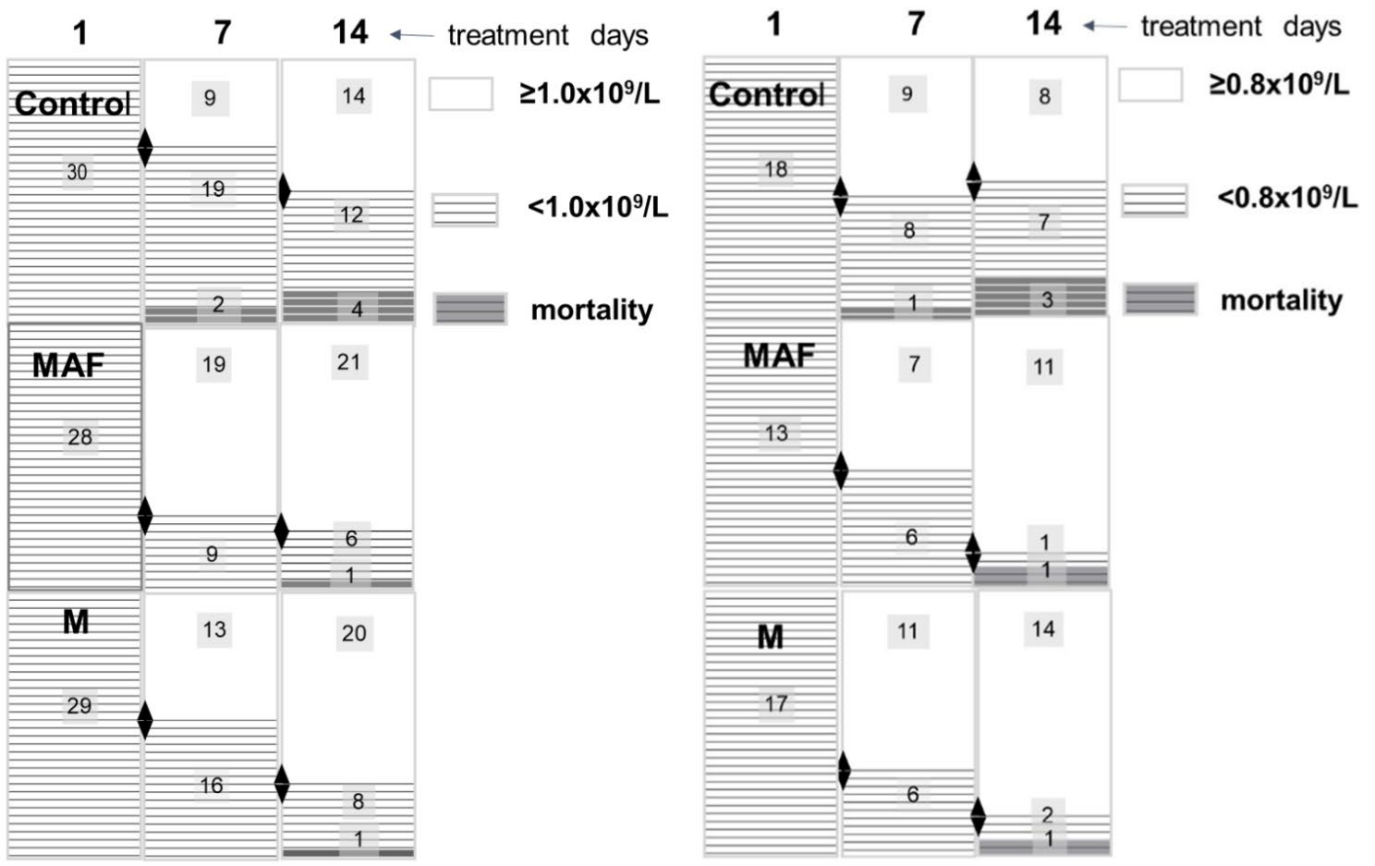
Distribution of patients in subgroups with ALC at enrolment below 1.0 × 10^9^/L (left) and in subgroups with ALC at enrolment below 0.8 × 10^9^/L (right) based on ALC recovery above these thresholds and mortality cases on Day 7 and Day 14* *Abbreviations* ALC, Absolute Lymphocyte Count; Control, Control subgroup; MAF, MAF subgroup; M, M subgroup.* Figure 2 visualizes the data presented in Table 8. This figure displays the distribution of the absolute number of patients in the lymphopenic subgroups of the study groups based on their ALC recovery above the indicated thresholds on Day 1 and Day 7 (whether achieved or not) and displays mortality cases

Figure 3 illustrates a higher trend in increasing mean ALC values in the lymphopenic subgroups under both study treatments compared to the control. In the subgroups with baseline ALC below 1.0 × 10^9^/L, mean ALC values (x10^9^/L) on days 1, 7, and 14 were as follows: MAF subgroup − 0.74, 1.17, 1.48; M subgroup − 0.69, 1.04, 1.51; control subgroup − 0.70, 0.88, 1.14. For those with baseline ALC below 0.8 × 10^9^/L: MAF subgroup − 0.57, 1.01, 1.29; M subgroup − 0.55, 0.90, 1.36; control subgroup − 0.58, 0.82, 0.97.

We analyzed changes in the proportion of patients with lymphopenia in study groups over the study treatment course, regardless of patients’ baseline ALC values (Table 8). The proportion of patients with an ALC lower than 1.0 × 10^9^/L on day 1 was 44.4%, 42%, and 41.7% in the MAF, M, and control groups, respectively. There was a significant reduction to 25.4% on day 7 in the MAF group and to 12.7%, 15.9%, and 19.4% in all the above groups, respectively, on day 14. On day 14, a significant reduction of the proportion of patients with profound lymphopenia (ALC < 0.8 × 10^9^/L) as compared to day 1 was seen in the MAF group (20.6% vs. 3.2%; *P* = 0.002) and the M group (27.0% vs. 7.9%; *P* = 0.005), while the control group did not show a significant reduction (25.0% vs. 15.3%; *P* = 0.229). The decreasing proportion of patients with profound lymphopenia from day 1 to day 14 was significantly greater in the MAF group as compared to the control (20.6–3.2% vs. 25–15.3%; *P* = 0.009).

Besides enhancing ALC recovery, the study agents demonstrated efficacy in preventing ALC depletion, a common occurrence during the course of COVID-19.The majority, 60 out of a total 69 events of any level of ALC depletion from the baseline level occurred on day 7. There were fewer ALC depletion incidences on day 7 in the MAF group, 15 out of 63 patients (23.8%), vs. 29 out of 72 patients (40.3%) in the control group (*P* = 0.045); and in the M group, 16 out of 69 patients (23.2%), vs. 29 out of 72 patients (40.3%) in the control group (*P* = 0.055); and similarly on day 14 in the MAF group, 16 out of 63 patients (25.4%), vs. 33 out of 72 patients (45.8%) in the control group (*P* = 0.019); and 20 out of 69 patients (29%) in the M group vs. 33 of 72 patients (45.8%) in the control group (*P* = 0.055). Among these, incidents of ALC depletion by ≥ 50% from the baseline level consisted of 7.9%, 5.8%, and 15.3% of patients in these groups respectively (Table 9).

**Table 5:** ALC and WBC in 10^9^ cells/L dynamic and some other characteristics in mortality cases

Subjects’*	Day 1		Day 7		Day 14		SpO2% on baseline	Hospital mortality day’
	WBC	ALC	WBC	ALC	WBC	ALC		
Control	6.37	1.45	11.45	0.59			93	12
Control	11.79	0.40					88	5
Control	6.73	0.75	7.26	0.52			89	9
Control	10.65	0.35	14.58	0.70	18.46	0.20	92	23
Control	8.48	0.64	8.48	0.85			92	10
Control	4.15	0.51	17.31	0.89	13.39	1.06	92	16
Control	9.79	0.85	17.95	1.10	23.33	0.79	92	15
Control	17.42	1.33					90	7
Control	5.41	2.58	8.33	4.25	19.46	9.09	90	17
Control	10.69	0.38	12.65	0.74	13.26	0.22	85	17
Control	4.80	0.96					91	3
MAF	19.19	0.68	6.82	1.22	8.63	0.91	93	17
MAF	9.92	0.44	11.77	0.53			90	9
M	4.92	0.56	17.16	0.95	13.94	0.71	92	13
M	6.78	0.53	7.93	0.80			88	9
M	8.64	0.90	5.44	0.49	9.67	0.51	92	34

*Abbreviations* WBC - White Blood Cells Count, ALC - Absolute Lymphocyte Count. * Control - Control group, MAF - MAF group, M - M group

**Table 6:** Change in median values of WBC and ALC over a two-week treatment period in the study groups

	Control **N = 72**		% from BL*	MAF **N = 63**		% from BL*	M N = 69		% from BL*	*P*-value					
										(vs. Control)		(vs. Day 1)			Group **time
	Median [95% CI]			Median [95% CI]			Median [95% CI]			MAF	M	Contr	MAF	M	
WBC 10^9^/L															0.726
Day 1	8.11	[7.16, 9.05]		7.60	[6.58, 8.61]		7.19	[6.22, 8.15]		0.470	0.183	-	-	-	
Day 7	9.07	[8.10, 10.03]		8.46	[7.44, 9.47]		7.87	[6.90, 8.83]		0.391	0.084	0.073	0.128	0.208	
Day 14	9.28	[8.30, 10.26]		7.94	[6.92, 8.96]		8.31	[7.33, 9.28]		0.064	0.169	**0.032**	0.541	**0.040**	
ALC 10^9^/L															0.801
Day 1	1.26	[1.09, 1.42]		1.12	[0.94, 1.30]		1.24	[1.07, 1.41]		0.261	0.876	-	-	-	
Day 7	1.40	[1.24, 1.57]	11%	1.41	[1.23, 1.58]	26%	1.44	[1.27, 1.61]	16%	0.972	0.748	**0.081**	**0.001**	**0.015**	
Day 14	1.73	[1.56, 1.90]	37%	1.69	[1.51, 1.86]	51%	1.79	[1.62, 1.96]	44%	0.729	0.640	**0.000**	**0.000**	**0.000**	

*Abbreviations* WBC, White Blood Cells Count; ALC, Absolute Lymphocyte Count; BL, baseline; Control, Control group; MAF, MAF group; M, M group,

*The level of increase, expressed as a percentage of the median ALC values on day 7 and day 14 in the study group compared to baseline

**Analysis Linear mixed model analysis with subjects as a random factor and time, group and their interaction (time*group) as a fixed factor

**Table 7:** Subgroups of patients in study groups with ALC below 1.0 × 10^9^/L and 0.8 × 10^9^/L on Day 1, and proportion of those among survivors whose ALC did not achieve recovery above these thresholds between Day 7 and Day 14

	Control		MAF		M		P-value				
							(vs. Control)		(vs. Day 1)		
							MAF	M	Control	MAF	M
**No. patients/%**	N*	%	N*	%	N*	%					
**Less 1.0 × 10** ^**9**^ **/L**											
**Day 1**	30/30	100	28/28	100	29/29	100			-	-	-
**Day 7**	19/28	67.9	9/28	32.1	16/29	55.2	**0,008**	0,358	**0,003**	**< 0.001**	**< 0.001**
**Day 14**	12/26	46.2	6/27	22.2	8/28	28.6	0,087	0,215	**< 0.001**	**< 0.001**	**< 0.001**
**Less 0.8 × 10** ^**9**^ **/L**											
**Day 1**	18/18	100	13/13	100	17/17	100			-	-	-
**Day 7**	8/17	47.1	6/13	46.2	6/17	35.3	0,973	0,534	**< 0.001**	**< 0.001**	**< 0.001**
**Day 14**	7/15	46.7	1/12	8.3	2/16	12.5	**0,025**	**0,046**	**< 0.001**	**< 0.001**	**< 0.001**

*Abbreviations* ALC, Absolute Lymphocyte Count; Control, Control subgroup; MAF, MAF subgroup; M, M subgroup

* 1. Data among of the patients with ALC less than 1.0 × 10^9^/L at enrolment: number of patients with ALC value less than 1.0 × 10^9^/L **/**number of survivors at the indicated time point

2. Data among of the patients with ALC less than 0.8 × 10^9^/L at enrolment: number of patients with ALC value less than 0.8 × 10^9^/L **/**number of survivors at the indicated time point

**Table 8:** Proportion of patients with ALC levels below 1.0 × 10^9^/L and 0.8 × 10^9^/L in study groups through Day 14

	Control *N* = 72		MAF *N* = 63		M *N* = 69		P-value					
							(vs. Control)		(vs. Day 1)			Group *time
							MAF	M	Contr	MAF	M	
No. patients/%	N	%	n	%	N	%						
Less 1.0 × 10^9^/L												0.654
Day 1**	30	41.7	28	44.4	29	46.0	0.800	0.979	-	-	-	
Day 7***	26	36.1	16	25.4	23	36.5	0.114	0.552	0.632	**0.023**	0.294	
Day 14***	14	19.4	8	12.7	10	15.9	0.198	0.328	**0.008**	**< 0.001**	**< 0.001**	
Less 0.8 × 10^9^/L												0.451
Day 1**	18	25.0	13	20.6	17	27.0	0.519	0.923	-	-	-	
Day 7***	16	22.2	10	15.9	12	19.0	0.271	0.376	0.804	0.492	0.298	
Day 14***	11	15.3	2	3.2	5	7.9	**0.009**	0.097	0.229	**0.002**	**0.005**	

*Abbreviations* ALC, Absolute Lymphocyte Count; Control, Control group; MAF, MAF group; M, M group

* Analysis Linear mixed model analysis with subjects as a random factor and time, group, and their interaction (time*group) as a fixed factor

** The number enrolled lymphopenic patients defined by having ALC lower than 1.0 × 10^9^/L and lower than 0.8 × 10^9^/L and percentage of these patients in the total number of patients in the study group

*** The number patients with ALC lower than 1.0 × 10^9^/L and with ALC lower than 0.8 × 10^9^/L and percentage of these patients in the total number of patients in the study group. This number includes both those ALC were not recover above the specified threshold from the previous visit and new patients whose ALC dropped below this threshold between the previous and current visit

### Adverse events

Adverse events were experienced by 43% of patients in the MAF group, 39% in the M group, and 56% in the control group; the difference in proportions between the MAF group and the control group and M group and the control group was not statistically significant (Table 9). Tolerability-related adverse events, that were more common in the control group, included nausea and headache. Serious adverse events were less common for both MAF and M groups (3 [5%] and 4 [6%] respectively) than in the control group (9 [13%]). All 15 deaths through day 29 (2 [3%] in the MAF group, 2 [3%] in the M group, and 11 [15%] in the control group) in 80% occurred in patients with initial lymphopenia, and none were attributed to any of the two investigated agents or standard care.

### Safety outcomes

**Table 9:** Adverse Event Summary occurring in Participants till day 29 term in study groups ^a^

Adverse events	Control *N* = 72		MAF *N* = 63		M *N* = 69		P-value (vs. Control)^d^	
							MAF	M
No. of events/% of total patients no.	N	%	n	%	n	%		
Any adverse event	40	55.6	27	42.8	27	39.1	0.169	0.064
Any grade ≥ 3 adverse events	7	9.7	5	7.9	5	7.2	0.770	0.765
Any serious adverse event	9	12.5	3	4.8	4	5.8	0.139	0.245
Discontinuation of treatment because of adverse event	NA		0		0			
Death day 14	6	8.3	1	1.6	2	2.9	0.121	0.276
Death day 29	11	15.3	2	3.2	2	2.9	**0.020**	**0.017**
**Adverse events occurring in > 5% of participants in any treatment group**								
Nausea	9	12.5	5	7.9	4	5.8	0.414	0.245
Headache	6	8.3	4	6.3	5	7.2	0.750	0.999
Diarrhea	2	2.8	3	4.8	2	2.9	0.664	0.999
**Laboratory abnormalities**								
**Hemoglobin decreased**								
Any level	18	25	19	30.1	15	21.7	0.564	0.694
8–10 g/dL					1	1.4		
7 to < 8 g/dL	1	1.4			1	1.4		
< 7 g/dL	2	2.8						
**Lymphocyte count decreased** ^**b**^								
Any level	33	45.8	16	25.4	20	29.0	**0.019**	0.055
On day 7	29	40.3	15	23.8	16	23.2	**0.045**	**0.032**
On day 7 and day 14	11	15.3	4	6.3	4	5.8	0.168	0.100
On day 14	4	5.6	1	1.6	4	5.8	0.371	0.999
≥ 50% from BL on day 7 and/or day 14	11	15.3	5	7.9	4	5.8	0.286	0.100
**ALT increase**								
Any level	30	41.7	22	34.9	32	46.4	0.480	0.613
< 2 times from BL	11	15.3	5	7.9	11	15.9	0.286	0.999
2 to 3 times from BL	6	8.3	9	14.3	9	13.0	0.289	0.421
> 3 times from BL	13	18.1	8	12.7	12	17.4	0.478	0.999
Grade 3 (> 5 to 10 times ULN)	1	1.4	1	1.6			0.999	0.245
**AST increase**								
Any level	14	19.4	15	23.8	14	20.3	0.675	0.999
< 2 times from BL	7	9.7	8	12.7	11	15.9	0.597	0.318
2 to ≥ 3 times from BL	3	4.2	5	7.9	3	4.4	0.472	0.999
> 3 times from BL	4	5.6	2	3.1			0.685	0.120
Grade 3 (> 5 to 10 times ULN)	1	1.4					1.000	1.000
**Creatinine increase** ^**ç**^								
Any level	24	33.3	11	17.5	14	20.3	**<0.001**	0.091
Grade 3 creatinine renal clearance decrease on 30% to < 50% from BL	3	4.2	1	1.6			0.623	0.245

*Abbreviations* AST, aspartate aminotransferase; ALT, alanine aminotransferase; BL, baseline levels; ULN, an upper limit of normal

a All safety analyses include data available for patients through day 29 for clinical data and in time points on day 1, day 7, and day 14 for laboratory data

b The number of participants with lymphocyte count decreased from BL on day 7 and restored to BL level on day 14, remained lower than the BL level on day 7 and day 14, and was found to decrease from BL level on day 14 only

ç The combined number of participants with blood creatinine increased or creatinine renal clearance decreased

d P-value: Fisher’s exact test [No correction for multiplicity]

## Discussion

This open-label, randomized, proof of concept, three-arm trial identified a new type of immune-modulating agent as beneficial in the treatment of hospitalized patients with non-critical COVID-19. Our overall findings were that a 14-day course of MAF capsules and M capsules were superior to control in the combined use with SOC treatment in hospitalized patients with COVID-19. All-cause mortality by day 14 was 1.6% in the MAF group, 2.9% in the M group, and 8.3% in the control group, and mortality by day 29 was 3.2%, 2.9%, and 15.3% in these three groups respectively. Fisher’s exact test estimates the reduction in mortality in the MAF group vs. control group by day 14 (*P* = 0.121) and by day 29 (*P* = 0.020); and in the M group vs. control group by day 14 (*P* = 0.276) and by day 29 (*P* = 0.046). Survivors on day 29 who received the study agents had a shorter time to basic improvement when they did not require any more supplemental oxygen than patients in the control group (median, 6 days in the MAF group compared to 8 days in the control group, *P* = 0.030; median, 6 days in the M group compared to 8 days in the control group, *P* = 0.006). Initial hospital stay was one day shorter for patients in the MAF group than those in the control group (median, 14 days vs. 13 days; *P* = 0.064) and similarly for patients in the M group than those in the control group (median, 14 days vs. 13 days; *P* = 0.017).

Patients receiving either of the study agents were more likely to have an improvement in the ordinal scale score. On day 14, 87.3% of patients in the MAF group and 86.9% of patients in the M group versus 73.6% of patients in the control group reached one of the primary recovery endpoints: when they did not require supplemental oxygen till being hospitalized or discharged from the hospital. Among 14-day recovery cohorts, the proportion of total discharged from the hospital was 63.4%, 66.6%, and 59.7% in the MAF group, the M group, and the control group respectively; the proportion of those discharged without limitations on their activities was significantly greater in the MAF group (55.4% vs. 29.2%; *P* = 0.03) and also in the M group (50.7% compared to 29.2%; *P* = 0.01).

Additional secondary endpoints supporting the findings of the primary outcome include MAF and M capsules use resulting in the prevention of respiratory deterioration seen on smaller events of new noninvasive ventilation or high-flow oxygen use in the MAF group compared to the control group (10.5% vs. 16.7%) and in the M group compared to the control group (6.5% vs. 16.7%). An even more notable difference was seen in the proportion of mechanical ventilation use which was lower in the MAF group than in the control group (3.2% vs. 12.5%; *P* = 0.061) and was significantly lower in the M group than in the control group (1.4% vs. 12.5%; *P* = 0.018).

Our data suggest that treatment with either of the study agents may decrease in-hospital mortality, by preventing the progression to more severe respiratory disease, as shown by the lower proportion of respiratory failures among patients in the MAF and M groups with subsequently a lower proportion of patients needing higher levels of respiratory support during the study. The benefit of recovery on MAF capsules and M capsules was fewer days of subsequent oxygen use, shorter length of initial hospital stay, and around twice decreasing the proportion of patients without limitations on their activities after discharge.

All included patients had confirmed lung involvement. Our results indicate that an enrolment lymphopenia of less than 0.8 × 10^9^/L imposed a multiplicative effect on the risk of mortality, therein the mortality on day 29, which consisted of 21% (10 out of 48 patients), which was nearly three times higher than on the whole intent-to-treat population; and it consisted of two thirds (10 out of 15) of the total mortality cases at this time point. The day 29 mortality in subgroups of those who had baseline ALC values lower than 0.8 × 10^9^/L was lower in MAF and M groups and consisted of 2 out 13 (15%) and 2 out 17 (12%) patients respectively versus 6 out of 18 (33%) patients in the control group. Therein the twice-lowering mortality rate in both intervention groups was linked to the earlier and greater ALC restoration seen in increasing mean ALC values (Fig. 3) and decreasing lymphopenia cases during the first two weeks of the study.

Earlier and greater ALC restoration in MAF and M groups was seen in 87 patients in the intent-to-treat population who had initial ALC lower than 1.0 × 10^9^/L, and it was most obviously seen in 48 of those who had more profound lymphopenia with baseline ALC lower than 0.8 × 10^9^/L.

Among the survivors in the subgroups of patients’ those had ALC lower than 1.0 × 10^9^/L at baseline, ALC exceeded the 1.0 × 10^9^/L level in 67.9%, 44.8%, and 32.1% of patients on day 7, and in 77.8%, 71.4%, and 53.8% of patients on day 14 day in lymphopenic subgroups of MAF in M and control group respectively.

At the end of the study treatment, on day 14, among the survivors in subgroups patients’ those had profound lymphopenia (baseline ALC lower 0.8 × 10^9^/L) ALC exceeded 0.8 × 10^9^/L in 91.7% and 87.5% patients in MAF and M subgroup respectively, compared to 53.3% of patients in the relative control subgroup. And the proportion of vulnerable patients in terms of mortality, those whose ALC remained not restored above the 0.8 × 10^9^/L level was 5.6 times less in the MAF subgroup and 3.8 times less in the M subgroup compared to the subgroup of control. The profound lymphopenia study cohort appeared to be the most responsive to both investigated treatments.

The effect of the study agents on lymphocyte count restoration was confirmed in the whole intent-to-treat population. The median of baseline ALC values was close to the low margin of the normal range in study groups; its first significant increase was seen earlier in the MAF and M groups on day 7 and then one week later in the control group. The level of increase was greater in both study groups, median ALC increased from baseline on day 7 and day 14 respectively − 26% and 51% in the MAF group, 16% and 44% in the M group versus 11% and 37% in the control group.

Both MAF capsules and M capsules are shown to prevent ALC depletion, especially severe ≥ 50% ALC decline from the baseline values. The incidences of any level of ALC depletion from baseline values occurred on or before day 14 in 25.4% of patients in the MAF group, 29% of patients in the M group, and 45.8% of patients in the control group; the incidents of ALC depletion by ≥ 50% consisted of 7.9%, 5.8%, and 15.3% of patients in these groups respectively.

Little was known about the pathogenesis of COVID-19 when the trial was designed in October 2020. Our initial expectations of the mechanism of action of the study agents were mainly based on anti-inflammatory effects via targeting of macrophages in the gut mucosa known to be able to down-regulate the systemic inflammatory response (see background section). However, in the intervention groups, there was no significant superiority over the control group in decreasing neither inflammatory biochemical marker as C reactive protein, and also on ferritin and lactate dehydrogenase levels. This could be attributed to the use of potent anti-inflammatory therapy as part of SOC in all three study groups.

However, unexpected stimulation of lymphopoiesis was revealed, which was most potent in conditions of profound lymphopenia. This is the first trial demonstrating that boosting the recovery of the low lymphocyte count and preventing it from further depletion is a promising approach to improve COVID-19 clinical outcomes. Study agents should also be clinically tested for their effectiveness in promoting lymphocyte count recovery in various viral infections, including mononucleosis, Ebola, influenza, measles, and viral hepatitis. Additionally, these agents can be evaluated for their potential use in managing of toxic drug side effects, cancer treatment, and long-term steroid therapy.

The lymphocyte phenotype distribution and molecular mechanisms of its recovery with the applied treatments deserve further investigation. In previous studies, it was shown that Saisei MAF-induced phagocytosis is accomplished with antigen processing. The lysing activity as judged by a reduction in pH and transition of antigens into phagolysosomes or lysosomes is followed by phagocytosis [3, 5, 6]. It can boost SARS-CoV-2 recognition and processing by macrophages and viral antigen presentation to lymphocytes. Hypothetically, this, on the background of controlling excessive inflammation, could be part of the mechanism of preserving lymphocyte functionality and numbers during COVID-19.

## Conclusion

Both study agents prevented ALC depletion and demonstrated improved ALC recovery in lymphopenia cases. This is considered one of the mechanisms for improving COVID-19 clinical outcomes which results in decreased mortality among lymphopenic patients. Our data showed that MAF capsules and M capsules were superior to SOC in decreasing respiratory deterioration, and mortality and shortening the time to recovery in adults who were hospitalized with non-critical COVID-19.

## Declarations

Numerous challenges were encountered during this trial planning and implementation. MAF capsules are considered to be a potential immunomodulator that increases antigen processing and the capacity of macrophages to resolve inflammation and modulate the mucosal immune response in the small intestine in conditions of acute COVID-19. Supporting this concept based on pre-clinical research, Saisei Pharma applied to the US FDA COVID-19 Scientific-Technical Triage for the evaluation of the rationale to study the efficacy of MAF capsules as a dietary supplement in the treatment of COVID-19. The study of this agent as a new drug was recommended, with the key points of the US FDA PIND 151,946 meeting from 16-Oct-2020 being to first conduct a small phase 2, proof-of-concept study to evaluate the safety and preliminary evidence of the efficacy of the product, with one of the following primary endpoints: (a) Mortality at a prespecified time point, (b) The proportion of subjects alive without needing mechanical ventilation using a pre-specified time point, (c) The proportion of subjects alive and free of respiratory failure (e.g., need for non-invasive or invasive mechanical ventilation, high flow nasal cannula oxygen, or ECMO) using a pre-specified time point. It was decided to first run the clinical trial of MAF and M capsules as dietary supplements in hospitalized non-critical COVID-19 patients using the U.S. FDA-recommended study design to have an initial proof of efficacy using the indicated endpoints. However, it became unfeasible to obtain ethical approval for a blinded study with dietary supplements. To overcome this issue an open-label clinical trial with the implementation of the U.S. FDA-recommended efficacy endpoints was initiated. To increase trial transparency, we applied to Ukraine’s government regulator asking for external monitoring. However, the response was that they were not providing this service for clinical trials of food supplements. This proof of concept trial was planned and performed at two Ukrainian clinical sites whereas, for the study team and study participants’ convenience, the study protocol and other materials were presented in Ukrainian. Training, site initiation visits, and monitoring visits were performed at both clinical sites via a site visit. The trial was implemented during a time when there was limited knowledge about COVID-19 and its treatment standards were just under development. Given the expected severity of the COVID-19 clinical course in hospitalized patients, there was special consideration regarding SOC to ensure that it was in line with WHO and local recommendations, and sufficient and equal for each study group.

Throughout the trial, we were able to enrol a patient cohort representing the Ukrainian population that was infected with SARS-CoV-2 and required hospitalization during that period. The first patient was enrolled on 27 October 2020 and the last patient was enrolled on 22 June 2021. The trial was stopped early because of dramatically declining hospitalized cases and enrolment. However, the statistical analysis of the 204 enrolled patients showed the significant superiority of adding both studied agents to SOC compared to SOC alone in hospitalized COVID-19 patients.

### Electronic supplementary material

Below is the link to the electronic supplementary material.

Supplementary Material 1

Supplementary Material 2

## Data Availability

Study data are available in the ISARIC COVID-19 RAPID Database and data supported the main study’ findings provided in supplementary file SaiseiCovUKR Statistical report 1.5 deidentified.
