# Supplementary material for: Effect of degalactosylated bovine glycoprotein formulations MAF and M сapsules on lymphopenia and clinical outcomes in hospitalized COVID-19 patients: a randomized clinical trial
Source: BMC Infect Dis. 2024 May 23;24:519. doi: 10.1186/s12879-024-09286-0 (PMC11112872; doi:10.1186/s12879-024-09286-0)
Supplement: Supplementary file 2 — Supplementary Material 2 [file 12879_2024_9286_MOESM2_ESM.docx]

**Randomization, as-treated population, data collection and performed statistical analysis**

This is an open-label, randomized, proof-of-concept, adaptive design clinical trial. This study reflects a US FDA PIND 151946 recommendation to conduct a small phase 2, proof-of-concept study to obtain evidence of MAF product efficacy in decreasing hospital mortality in non-critical Covid-19 patients. The trial included adult patients hospitalized with non-critical Covid-19 who received for 14 days degalactosylated bovine glycoproteins formulations of either MAF capsules (MAF group) or M capsules (M group) ​​in addition to standard of care (SOC) or SOC only (control group). Eligible patients were randomly assigned in a 1:1:1 ratio to receive either SOC only (control group), or MAF capsules (MAF group) or M capsules (M group) in addition to SOC.

**Randomization**

Randomization list is performed for each clinical site separately using [Random Allocation Software](https://random-allocation-software.software.informer.com/) 2.0. Sequential Block-permuted randomization is used to assign eligible participants in a 1:1:1 ratio. Patients enrolled in this trial were first grouped into strata by age: less than 60 years old and 60 years and older. Then within each of these two strata, patients were assigned to treatment groups in 1:1:1 according to separate for each strata randomization list. After the patient was enrolled in the study, the recruiting doctor contacted the designated center by phone and provided the information about the name of the clinical site and the enrolled patient’s age. Based on this information recruiting doctor was informed of the patient’s assignment to the proper treatment group.

**Study Data collection**

The two study clinical sites were implemented within a network of hospitals that collect data using the ISARIC-World Health Organization Clinical Characterization Protocol and data tools for COVID-19 patients. Clinical sites adhered to the standardized in ISARIC COVID -19 study laboratory units, clinical and laboratory data collection algorithms, gathering data through an international electronic data capture system, contributing to the formation of global databases with prospectively collected clinical data on individuals hospitalized with COVID-19 (https://isaric.org/). These electronic databases facilitated remote, real-time monitoring of the captured study data.The electronic Case Report Form (eCRF) for study participants includes various modules: the enrollment module, day 1, 7, 14, and 29 study treatment visits modules, intensive care treatment, and discharge and two post treatments follow up modules. The reported during acute COVID-19 treatments data encompass a wide range of information, including signs and symptoms, pre-existing comorbidities, anthropometric data, vital signs, chronic and acute treatments, study treatments, complications, laboratory data, dates of hospitalization and discharge, mortality, and vaccination status. In addition to the eCRF modules, a paper version CRF was utilized. It included all the modules listed above, along with the daily monitoring of vital signs (blood pressure, respiratory rate, body temperature, oxygen saturation in capillary blood (SpO2%)), and daily changes in the COVID-19 WHO 8 score ordinary scale, treatment tolerability, and adverse reactions.

**Interim statistical analysis**

Since there were many unknowns in the results, the study was designed to conduct an interim analysis of the first 200 randomized participants to determine whether the study was validly or invalidly terminated. The interim analysis aimed to obtain preliminary data on the safety and efficacy of MAF and M capsules in decreasing the mortality of hospitalized Covid-19 patients. Where no interest in further study development was prespecified if the superiority in decreasing hospital mortality rate in both study groups will consist of 5% or less compared to the control.

In fact, the interim analysis included patients who were enrolled before the enrolment was interrupted in June 2021 due to dramatically declining hospitalized cases. The as-treated population at this time point included 204 patients who received the assigned treatment (63 assigned to the MAF group, 69 to the M group, and 72 to the control group). All analyses on the as-treated population were conducted according to the randomly assigned treatment where the two study groups were compared to the control and changes in the laboratory data were also analyzed on days 7 and 14 compared to baseline level.

The analysis of the data until study day 29 showed that MAF capsules and M capsules were superior to SOC in decreasing mortality and events of respiratory deterioration which required mechanical ventilation. The superiority of these crucial study points achieved adequate statistical power. The results of the interim analysis indicate that the study has been terminated as effective.

**Subgroups analysis:** This primary analysis showed decreased mortality in both study subgroups linked with greater ALC restoration in these groups. In this regards, the two weeks change (day 1, 7 and 14) in ALC values were analysed in subgroups of patients who had an enrolment lymphopenia with cut-off values of baseline ALC lower than 1.0×10^9^ cells/L and lower than 0.8×10^9^ cells/L. The proportions of patients in study groups with ALC levels lower than 1.0x10^9^/L and lower than 0.8×10^9^ cells/L on day 1, day 7, and day 14 were also analysed regardless of the patient’s baseline ALC value.

# All statistical analyses performed by independent statistician, Kyoto Medical Center, National Hospital Organization, Clinical Research Institute, Kyoto, Japan.

**Reason of the slight disbalance in the number of enrolment patients between study groups**

There were two coincided-in-time issues our study faced during May, and June 2021. Where the first was a delay in study products supply from Japan due to Covid-19 carnitine measures and the second issue was progressively declining hospitalized patients in the study clinical sites. In this situation, it was decided to keep enrolling study subjects in accordance with the actual randomization schedule under the following rule: if the patient was assigned to one of the study groups where the study product was unavailable, that patient was assigned to the control group. This study run in a frame of the global ISARIC Covid-19 study and keeping enrolling study subjects till Covid-19 patients are available was our goal as the ISARIC study contributors. In the Luhansk region Covid-19 hospital there were four hospitalized with Covid-19 on the middle of June 2021 and no patients at the end of the month.
